# Arms and the Man: The Problem of Symmetric Growth

**DOI:** 10.1371/journal.pbio.1000477

**Published:** 2010-09-07

**Authors:** Lewis Wolpert

**Affiliations:** Department of Cell and Developmental Biology, University College, London, United Kingdom; University of California Irvine, United States of America

The external features of our bodies are specified in the embryo and then grow for some 16 years, yet many are remarkably symmetrical. Just consider how similar in size and shape your two ears are. And if you extend your arms, you will likely find that they, too, are similar in length, even though they grew independently from tiny buds in the embryo. Their length matches with an accuracy of about 0.2% yet there is no known communication between the limbs during growth. You'll find the same holds for your two forefingers as it does for the size of internal body organs such as the kidneys and lungs. How is such coordination achieved? While we have a reasonably good understanding of how our limbs grow, we know relatively little about how their growth is so reliably controlled.

## Mechanisms of Growth Control

Organ size in animals is determined by both intrinsic developmental programs and by extracellular factors that stimulate or inhibit growth, but the relative importance of these two mechanisms in different organs varies a good deal [1],[2]. Consider, for example, the spleen. When multiple fetal spleens are grafted into an embryo, each spleen grows much smaller than normal, so that the final total mass of the spleens is equivalent to one normal spleen. This indicates that growth of the spleen involves an extrinsic mechanism. Indeed, the spleen uses negative feedback mechanisms to regulate its final size by secreting some (as yet unknown) factors that inhibit growth. Upon reaching a certain size, the concentration of inhibitory factors is sufficient to stop further growth. We also know that extrinsic mechanisms control liver size, since the liver can regenerate to its normal size when a piece is removed; this, too, involves some sort of negative feedback.

By contrast, the thymus is controlled by intrinsic growth control mechanisms. We know this because when multiple fetal thymus glands are transplanted into a developing mouse embryo, each one grows to full size, indicating a primarily intrinsic control. Another illustration of an intrinsic growth program comes from grafting limb buds between large and small species of salamanders of the genus *Ambystoma*. A limb bud from the larger species grafted onto the smaller species initially grows slowly, but eventually reaches its normal size, which is much larger than any of the host's limbs.

## Distinguishing Growth and Symmetry

The development of symmetry in limb buds in the embryo appears to depend on the presence of positive signalling feedback loops during limb bud growth [3]. However, these mechanisms are quite different from those that control the growth of the limb, which is due to bone growth at the growth plates at the proximal and distal ends of each long bone. Thus, the growth of the long bones of the arm—humerus, radius, and ulna—and the phalanges of the digits are responsible for ultimate limb length.

Only an intrinsic growth programme can explain the control of limb growth as the growing region in the bones because there is no evidence that the growth plates have a means of sensing how much the bone has grown. While, in principle, bones could secrete circulating factors affecting growth in the plates, there is no evidence for this. Moreover, in growth plate–transplantation experiments, the growth rate of the transplanted growth plate depends on the age and hence the size of the donor animal, not on that of the recipient.

**Figure 1:**
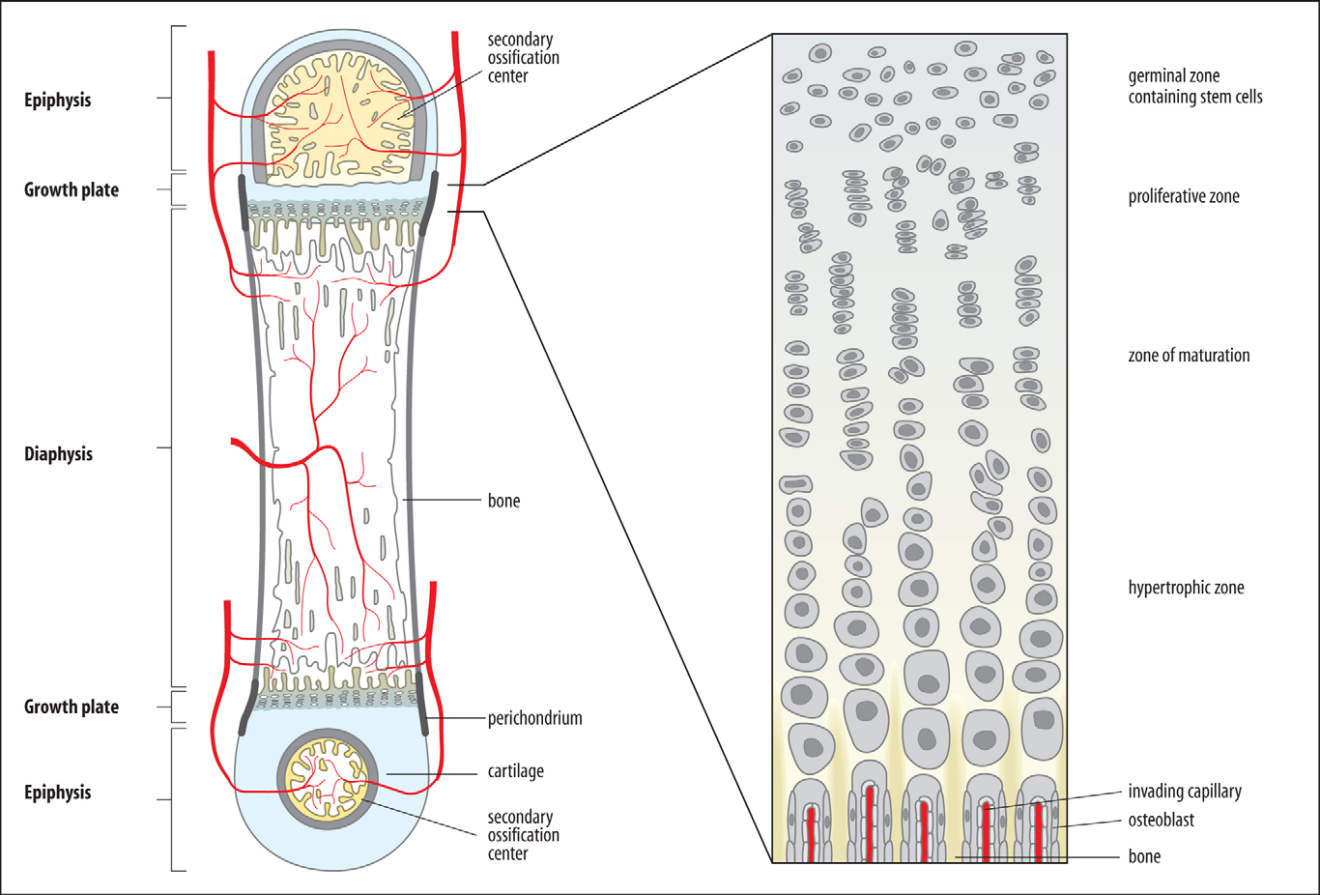
The long bones of vertebrate limbs increase in length by growth from cartilaginous growth plates. The growth plates are cartilaginous regions that lie between the epiphysis of the future joint and the central region of the bone, the diaphysis. In the figure, bone has already replaced cartilage in the diaphysis, and more bone is being added at the growth plates. Within the growth plates, cartilage cells multiply in the proliferative zone, then mature and undergo cell enlargement and extend the bone. They are then replaced by bone [1]. (Image: Oxford University Press)

The growth plates extend the bone but they remain about the same size for many years, as the cartilage cells they produce are replaced by bone (Figure 1). In the growth plates, the cells behave differently in three main regions. At the top end near the epiphysis are the stem cells, then comes a zone of cell proliferation, followed by the cells developing into columns of cartilage cells (chondrocytes), where the cells undergo hypertrophy, increasing their size from 4-to 10-fold [4]. At the bottom end, the cartilage cells undergo programmed cell death (or apoptosis) and are replaced by bone. Thus, cell proliferation leads to more hypertrophic chondrocytes, which are replaced by bone. This sequence pushes the growth plate away from the bone region and ultimately increases the length of the bone.

## How Does Symmetry Emerge Despite Vast Differences in Cell Number, Rates of Growth, and Size?

A high growth rate in limbs is observed from fetal life, with a rapid deceleration up to about three years of age. The second phase is characterized by a period of lower, slowly decelerating growth velocity up to puberty. The last phase, puberty, is characterized by an increased rate of longitudinal growth until the age of peak height velocity has been reached. Then, growth velocity rapidly decreases due to growth plate maturation in long bones and spine, leading to growth plate fusion and cessation of longitudinal growth. Growth continues through childhood but gets slower until there is a spurt at adolescence, mainly due to an increase in the size of the hypertrophied cells, after which growth ceases and the growth plate fuses and disappears. At a single stage during growth, plates in different bones can elongate at rates that differ by a factor of seven or even more. Even the growth plates at the ends of the same bone can elongate at significantly different rates, again consistent with an intrinsic programme. Fusion of the growth plate is a result, and not the cause of ceased growth. When growth stops, the plate disappears. Epiphyseal fusion is triggered when the proliferative potential of growth plate chondrocytes is exhausted; and estrogen does not induce growth plate ossification directly, but accelerates the programmed senescence of the growth plate, thus causing earlier proliferative exhaustion and consequently earlier fusion.

The number of cells in a column is of the order of 40. Cells can be produced at rates of over 10,000 a day, yet the number of cells needs to be identical (or very nearly so) on both limbs for years. In a typical rat growth plate eight chondrocytes leave each growth plate column each day and are replaced by cells at the top of the column. Thus, increase in length of the bone, which can occur with columns keeping the same length, is due mainly to hypertrophy and cell proliferation. The rate of increase in length due to a growth plate is equal to the rate of new cell production for each column in the proliferative zone multiplied by the mean height of the hypertrophied cells. Different growth plates in the limb provide growth at different rates, and this can be due to differences in the size of the proliferative zone and the rate of cell proliferation, as well as the degree of cell enlargement when the cells hypertrophy. In the rat proximal tibia plate, the number of new cells per day is 16,400 with a standard deviation of 5,850, the cell cycle time is roughly 30 hours, and height of the columns is about 620 microns [5].The growth rate was 400 microns per day. Yet, in spite of these large numbers and their variance, the growth is highly reliable and the same on both left and right limbs.

The lengths of proliferative columns in individual bones are controlled by the growth factors PTHrP and Indian hedgehog (Ihh) [6]. The sharpness of the transition between proliferating and hypertrophic cells may be increased by local feedback between Ihh and PTHrP production. Ihh stimulates chondrocyte proliferation directly and, through stimulation of PTHrP synthesis, determines the distance from the end of the bone at which chondrocytes stop proliferating and undergo hypertrophic differentiation. PTHRrP, and Ihh form a positive feedback loop. PHRP is produced in the proliferative zone and stimulates proliferation and blocks Ihh synthesis. When the cells are away from the proliferative zone, they then synthesize Ihh, which diffuses back and stimulates proliferation. FGF signalling shortens proliferative columns, both by decreasing chondrocyte proliferation directly and by suppressing Ihh expression. BMPs act at each of these steps in a manner opposite to that of FGFs. The determinants of the boundaries between the three main regions and the polarity of the columns are not known.

## Could Hormones Play a Role?

The major systemic hormones that regulate longitudinal bone growth during childhood are GH and IGF-1, thyroid hormone (T_3_ and T_4_), and glucocorticoids (GC), whereas during puberty the sex steroids (androgens and estrogens) contribute a great deal to this process [7]. Growth hormone (GH) and insulin-like growth factors (IGFs) are potent stimulators of longitudinal bone growth and in both boys and girls; estrogen is the main determinant for the puberty-associated phenomena related to longitudinal growth and bone quality. These hormones could help coordinate growth across the body.

According to the current view, growth ceases because the cartilage cells have a finite growth potential [8], that is, they have an intrinsic growth programme. In the human embryo the linear growth rate is 20 times greater than that in mid childhood, when there is a significant decline. And, apart from the increase at puberty, the proliferative cells then become senescent. Despite these changes in growth rate, the bones remain similar size on both sides of the body. There was a view that this trajectory was controlled by circulating hormones, possibly a neuroendocrine factor, but this is clearly not the case, and there is no evidence for extrinsic controls. There is a progressive decline in growth rate with age, and transplanting a growth plate to a younger or older organism does not change the bone's growth rate, again consistent with intrinsic control. If growth is delayed by chemical treatment, the plate will then grow more rapidly for a short period when the treatment is removed, and this is known as catch up growth. This shows that timing is related to cell proliferation. If chondrocyte stem cells in the plate have a limited proliferative capacity, then this could determine when growth ceases. It is possible that growth inhibitors very slowly accumulate in the growth plate and so determine when growth ceases. Another possibility is that the stem cells have a mechanism for counting how many times they have divided, but it seems telomeres, which can control senescence in cells, are not the basis. Oestrogen (which circulates in the body) is, however, involved in growth plate closure and acts by speeding up senescence. Its role might be to ensure that all the growth plates close at about the same time [8].

## What Experiments Might Help Us Solve the Problem?

Given the complex interactions and signals in the growth plate, it is all the more remarkable that the intrinsic growth programmes of the different growth plates on the two sides of the body manage to produce arms of the same length with such precision and reliability. It's possible that the large number of cells in a growth plate favour reliable growth by reducing any effect of small differences in cell behaviour. One might test this possibility by running computer simulations of the growth plate to see whether the large number of cells in a growth plate would yield a reliable and consistent growth, in spite of any small variations in cell behaviour.

It would also be helpful to compare the cell dynamics in growth plates on left and right limbs to determine just how similar they are. Are the cell cycle times and number of dividing cells the same? Are the column lengths identical? Are the sizes of the three main regions along the columns the same? If cell proliferation in a growth plate is blocked for any period of time, do the bones reach the same final length on both sides? If some of the cells are killed does the bone grow shorter? And if a growth plate is replaced by a younger plate, does the length of the bone end up longer than that on the other side? As we investigate these questions and gain a better understanding of the signals controlling limb growth and size, we will, in turn, elucidate the intrinsic growth programme that endows us with remarkably symmetrical limbs. Solving this problem would provide major insights into growth control and will no doubt keep us busy seeking its solution for a good long while.
